# Supplementary material for: Enhancement of single upconversion nanoparticle imaging by topologically segregated core-shell structure with inward energy migration
Source: Nat Commun. 2022 Oct 7;13:5927. doi: 10.1038/s41467-022-33660-8 (PMC9546905; doi:10.1038/s41467-022-33660-8)
Supplement: Supplementary file 1 — Supplementary Information [file 41467_2022_33660_MOESM1_ESM.pdf]

Supplementary Information

Enhancement of single upconversion nanoparticle imaging by topologically segregated core-shell structure with inward energy migration

Yanxin Zhang[1,&], Rongrong Wen[1,&], Jialing Hu[1], Daoming Guan[1], Xiaochen Qiu[1], Yunxiang Zhang[1*], Daniel S. Kohane[2*], Qian Liu[1*]

[1] Department of Chemistry and Shanghai Key Laboratory of Molecular Catalysis and Innovative Materials, Fudan University, Shanghai 200438, China.

[2] Laboratory for Biomaterials and Drug Delivery, Division of Critical Care Medicine, Children's Hospital Boston, Harvard Medical School, 300 Longwood Avenue, Boston, MA 02115, USA.

[&]These authors contributed equally to this work.

*Correspondence and requests for the materials should be addressed to Y.Z. (zyx@fudan.edu.cn), D.S.K. (daniel.kohane@childrens.harvard.edu), or Q.L. (qianliu@fudan.edu.cn)

Supplementary Methods

Materials: All starting materials were obtained from commercial supplies. The salt crystals, $LuCl_3{\cdot}6H_2O$, $YbCl_3{\cdot}6H_2O$ and $ErCl_3{\cdot}6H_2O$ were purchased from Aladdin. NH_4F, NaOH, ethanol and cyclohexane were purchased from Sinopharm Chemical Reagent Co., Ltd. Octadecene (ODE) (>90%) and oleic acid (OA) (>90%) were purchased from Sigma-Aldrich. All chemical reagents of analytical grade were used directly without further purification.

Synthesis of the core of UCNPs

We used a typical solvent thermal method to synthesize all the UCNPs[1]. 10 mL of octadecene (ODE), 10 mL of OA, 0.8 mL $LuCl_3{\cdot}6H_2O$ aqueous solution (1.0 mol/L) and 0.2 mL $ErCl_3{\cdot}6H_2O$ aqueous solution (1.0 mol/L) were added into a 100 mL three-neck flask. The mixture was heated to 160°C for about 50 min under N_2 flow to remove the water. After that, it was cooled down to 110°C, then adding 2.03 g NaOA and 0.34 g NH_4F into the mixture and stirring for 30 min to dissolve the salts. Subsequently, the solution was heated to 300 ºC and maintained for 50 min at argon atmosphere. After the solution was cooled to room temperature, the mixture of ethyl alcohol and cyclohexane was poured into, and the resultant mixture was separated via centrifugation of 16099× g for 10 min. The products were collected and washed with ethanol and cyclohexane three times. Finally, all the samples were dispersed in 10 mL cyclohexane. Other core samples were synthesized by similar procedure only changing the corresponding rare-earth metals.

Synthesis of the core-shell of UCNPs

8 mL of octadecene (ODE), 3 mL of OA and 0.3 mL $YbCl_3 \cdot 6H_2O$ aqueous solution (1 mmol/mL) were loaded into a 100 mL three-neck flask, then heated to 160°C under N_2 flow to remove the water. After that, the mixture was cooled down to room temperature, 0.03 g NaOH and 0.0417 g NH_4F dissolved in 3.75 mL methanol and 3 mL previously prepared $NaLu_{0.8}Er_{0.2}F_4$ core in cyclohexane were added into the flask and stirred for 30 min at room temperature. Then the mixture was heated to 120°C and keep another 30 min. Subsequently, the solution was heated to 290°C and maintained for 30 min under argon atmosphere. Following by cooling to room temperature, the resultant solution was washed with 10 mL ethyl alcohol and 5 mL cyclohexane and separated via centrifugation of 16099× g for 10 min. The products were collected and washed with cyclohexane and ethanol (1:1, v/v) three times. Finally, all the samples were dispersed in 10 mL cyclohexane. Other core-shell samples were synthesized by similar procedure only changing to the corresponding rare-earth metals.

Synthesis of the core-shell-shell of UCNPs

The core-shell-shell UCNPs were obtained by growing another inert shell on previously prepared core-shell UCNPs. The procedure is similar with that of synthesizing core-shell UCNPs only replacing $NaLu_{0.8}Er_{0.2}F_4$ core with $NaLu_{0.8}Er_{0.2}F_4$ @$NaYbF_4$ core-shell UCNPs, and $YbCl_3 \cdot 6H_2O$ with $LuCl_3 \cdot 6H_2O$. Other core-shell-shell UCNPs were obtained by similar procedure only changing to their corresponding rare earth metals.

Synthesis of UCNPs@dSiO$_2$

UCNP@dSiO$_2$ was synthesized via water-in-oil reverse microemulsion[2]. 1 g Igepal CO-520 was dispersed in 10 mL of cyclohexane and sonicated for 5 min. 0.5 mL UCNPs cyclohexane solution was then added into the mixture and stirred for 3 hours. Subsequently, 50 μL of ammonia (30%) was added dropwise and stirred for 24 hours. Last, 5 μL of tetraethyl orthosilicate (TEOS) sonicated 30 min with 200 μL cyclohexane was slowly introduced into the reaction system. After keeping magnetic stirring for 24 hours, the resulting products were precipitated through adding acetone and then washed three times with ethanol and dispersed in 10 ml of deionized water (DI H_2O).

Nanoparticle's characterization

The particle size and morphology were character by TEM (HT7800) at an acceleration voltage of 100 kV. STEM and high-resolution TEM imaging were carried out in a FEI Tecnai G2 F20 S-Twin TEM at an acceleration voltage of 200 kV. The crystal structure and phase of samples were achieved Bruker D2 PHASER (Cu Kα radiation, $\lambda = 1.54056$ Å). The upconversion luminescence properties accepted from FLS 1000 spectrofluorometer accompanied with external 980-nm diode laser (Changchun New Industries Optoelectronics Technology Co., Ltd.). And the lifetime was also recorded from FLS 1000 spectrofluorometer equipped with external 980-nm diode laser and the pulse width ≤ 5 ms. The ICP data was measured using the PE-8000 inductively coupled plasma emission spectrometer.

Monte Carlo Simulations[3]

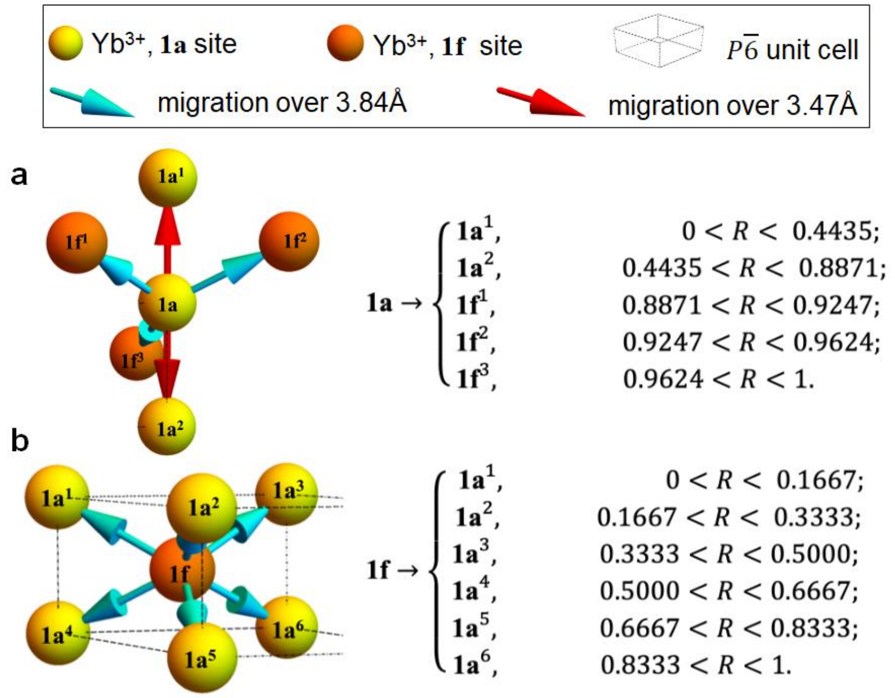

Supplementary Figure 1. Migration decision illustrated and manifested by random number $\boldsymbol{R} \in [\mathbf{0}, \mathbf{1}]$. (a) migration out of **1a** lattice site to five **1a** and **1f** sites with unequal probabilities; (b) migration out of **1f** lattice site to six sites with equal probability.

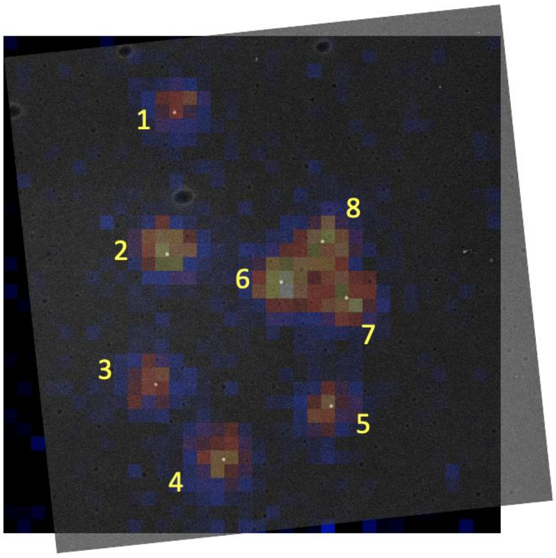

Supplementary Figure 2. Overlayed SEM and wide field images with N(N=8) numbered nanoparticles for 2D polynomial mapping.

To quantitatively analyze how the positions and/or distances in the wide field luminescence image match those in the SEM image, we did 1st degree 2D polynomial mapping[4] using the following equations.

$$\hat{\mathbf{x}}_{\mathbf{WF}} = p_{00} + p_{01}\mathbf{x}_{\mathbf{SEM}} + p_{10}\mathbf{y}_{\mathbf{SEM}} + p_{11}\mathbf{x}_{\mathbf{SEM}}\mathbf{y}_{\mathbf{SEM}} \quad (1)$$

$$\hat{\mathbf{y}}_{\mathbf{WF}} = q_{00} + q_{01}\mathbf{x}_{\mathbf{SEM}} + q_{10}\mathbf{y}_{\mathbf{SEM}} + q_{11}\mathbf{x}_{\mathbf{SEM}}\mathbf{y}_{\mathbf{SEM}} \quad (2)$$

All 8 nanoparticles' fitted positions in the wide field image and corresponding SEM image were used to generate the mapping coefficient matrixes P, Q.

$$\mathbf{P} = \begin{pmatrix} p_{00} & p_{10} \\ p_{01} & p_{11} \end{pmatrix} = \begin{pmatrix} \mathit{447.39450} & \mathit{-0.19931231} \\ \mathit{0.90278465} & \mathit{8.8982277e\text{-}005} \end{pmatrix} \quad (3)$$

$$\mathbf{Q} = \begin{pmatrix} q_{00} & q_{10} \\ q_{01} & q_{11} \end{pmatrix} = \begin{pmatrix} \mathit{-187.35820} & \mathit{1.0277292} \\ \mathit{0.096447140} & \mathit{-1.3890537e\text{-}005} \end{pmatrix} \quad (4)$$

The registration error for each nanoparticle can be estimated by subtracting registered position from the fitted position in the referencing wide field image.

$$\delta_{reg} = \sqrt{(\hat{\mathbf{x}}_{\mathbf{WF}} - \mathbf{x}_{\mathbf{WF}})^2 + (\hat{\mathbf{y}}_{\mathbf{WF}} - \mathbf{y}_{\mathbf{WF}})^2} \quad (5)$$

Nanoparticles #1~#5 exhibit registration errors ranging from 7.5 to 19 nm with a median of 9.8 nm, which is ~1/30 of the FWHM of the optical point spread function. Nanoparticle #6, #7 and #8 possess registration errors of 37, 42 and 25 nm respectively, which were ~1/10 of the FWHM, not bad considering their overlapping PSFs. An averaged registration error of ~24 nm for all 8 nanoparticles investigated can be estimated by the following equation.

$$\bar{\delta}_{reg} = \sqrt{\frac{\sum_{i=1}^{N}\left[\left(\hat{\mathbf{x}}_{\mathbf{WF,i}} - \mathbf{x}_{\mathbf{WF,i}}\right)^2 + \left(\hat{\mathbf{y}}_{\mathbf{WF,i}} - \mathbf{y}_{\mathbf{WF,i}}\right)^2\right]}{N}} \quad (6)$$

To summarize, the positional discrepancies between the fluorescence image and SEM micrograph is estimated to be as small as half the size of a nanoparticle (~8 nm) which is well within expected experimental errors and also an impressive one considering the registration error is merely a fraction of the size of a nanoparticle.

Upconversion quantum yield (UCQY) measurement:

The methods were adapted from the design of Veggel et al[5]. We perform the measurements from Edinburgh Instruments LFSP920 luminescence spectrometer modified with NIR PMT (HAMAMATSU, C9940-02, No. CA0142)and tested with an integrating sphere. The 120W/cm^2 excitation power density was chosen and un-doped $NaYF_4$ UCNPs were chosen as the reference sample to obtain absolute upconversion quantum yield. The UCQY value was calculated through the following equation[6] where the absolute UCQY is determined by first acquiring visible photons emitted (N_{em}), which is done by measuring the integrated emission intensity ($I_{Sample,}$ 400 nm to 700 nm), and dividing its value by the 980 nm photons absorbed (N_{abs}). The latter is subtracting the intensity of the excitation beam after propagating through the sample under review (T_{Sample}) from the intensity of the beam after passing through an equivalent undoped reference sample ($T_{Reference}$), possessing similar scattering properties.

$$\mathrm{UCQY} = \frac{\text{visible photons emitted}}{\text{980 nm photons absorbed}} = \frac{N_{em}(\lambda_{ex})}{N_{abs}(\lambda_{ex})} = \frac{I_{Sample}}{T_{Reference} - T_{Sample}} \quad (7)$$

Supplementary Table 1. Inductively coupled plasma atomic emission spectroscopy results of inside-out Yb@$Lu_{0.8}Er_{0.2}$@Lu, outside-in $Lu_{0.8}Er_{0.2}$@Yb@Lu, and local $Yb_{0.5}Lu_{0.4}Er_{0.1}$@Lu UCNPs.

Sample	Element	Percent (%)[1]	Standard deviation [2]
Yb@$Lu_{0.8}Er_{0.2}$ @Lu (inside-out)	Er	5.6800	0.0265
	Lu	60.7067	0.2589
	Yb	33.6100	0.2707
$Lu_{0.8}Er_{0.2}$@ Yb@Lu (outside-in)	Er	5.5100	0.0600
	Lu	65.2567	0.1680
	Yb	29.2367	0.1358
$Yb_{0.5}Lu_{0.4}Er_{0.1}$@Lu (local)	Er	6.1233	0.1002
	Lu	61.7600	0.3606
	Yb	32.1167	0.2950

[1] The percent value is the average of three parallel experiments.

[2] This is the standard deviation of three parallel experiments.

Supplementary Table 2. Lifetime results of the inside-out Yb@$Lu_{0.8}Er_{0.2}$@Lu, outside-in Lu0.8Er0.2@Yb@Lu, and local $Yb_{0.5}Lu_{0.4}Er_{0.1}$@Lu UCNPs.

Exciting wavelength	**541 nm**					**654 nm**				
	τ_1 (μs)	τ_2 (μs)	B_1	B_2	τ_{avg}* (μs)	τ_1 (μs)	τ_2 (μs)	B_1	B_2	τ_{avg}* (μs)
$Lu_{0.8}Er_{0.2}$@ Yb@ Lu (outside-in)	99.28	445.50	3.28	0.16	**161.58**	118.44	490.18	3.71	0.14	**168.74**
Yb0.5Lu0.4Er0.1 @Lu (local)	29.58	368.04	72.16	0.04	**31.78**	388.81	61.59	0.03	8.89	**68.90**
Yb@ $Lu_{0.8}Er_{0.2}$ @Lu (inside-out)	200.63	40.10	0.22	21.73	**47.79**	318.45	51.24	0.06	13.08	**58.81**

$$*\tau_{avg} = \frac{\sum B_i \tau_i^2}{\sum B_i \tau_i}$$

Supplementary Table 3. Lifetime of UCNPs, which was excited under $100 W/cm^2$ 980 nm laser and measured for 541 nm and 654 nm emissions.

Nanoparticle	541 nm emission (μs)	654 nm emission (μs)
$Lu_{0.95}Er_{0.05}$@Yb@Lu	114.38	105.51
Yb@$Lu_{0.95}Er_{0.05}$@Lu	85.39	69.04
$Yb_{0.5}Lu_{0.475}Er_{0.025}$@Lu	52.52	72.96
$Lu_{0.9}Er_{0.1}$@Yb@Lu	151.41	108.93
Yb@$Lu_{0.9}Er_{0.1}$@Lu	72.87	66.36
$Yb_{0.5}Lu_{0.45}Er_{0.05}$@Lu	94.61	101.50
$Lu_{0.8}Er_{0.2}$@$Yb_{0.75}Lu_{0.25}$@Lu	125.86	148.67
$Lu_{0.8}Er_{0.2}$@$Yb_{0.5}Lu_{0.5}$@Lu	108.61	126.88
$Lu_{0.7}Er_{0.3}$@Yb@Lu	132.97	137.94
Yb@$Lu_{0.7}Er_{0.3}$@Lu	81.54	43.95
$Yb_{0.5}Lu_{0.35}Er_{0.15}$@Lu	81.32	64.56
$Y_{0.9}Er_{0.1}$@Yb@Y	201.30	178.10
Yb@$Y_{0.9}Er_{0.1}$@Y	45.24	61.08
$Yb_{0.5}Y_{0.45}Er_{0.05}$@Y	56.71	66.17

Supplementary Table 4. The summary of UCQY for Lu-based samples.

Nanoparticle	Type	UCQY
$Lu_{0.95}Er_{0.05}$@Yb@Lu	Outside-in	1.6±0.4%
Yb@$Lu_{0.95}Er_{0.05}$@Lu	Inside-out	0.4±0.2%
$Yb_{0.5}Lu_{0.475}Er_{0.025}$@Lu	Local energy transfer	0.8±0.4%
$Lu_{0.9}Er_{0.1}$@Yb@Lu	Outside-in	1.9±0.4%
Yb@$Lu_{0.9}Er_{0.1}$@Lu	Inside-out	0.4±0.2%
$Yb_{0.5}Lu_{0.45}Er_{0.05}$@Lu	Local energy transfer	0.7±0.3%
$Lu_{0.8}Er_{0.2}$@Yb@Lu	Outside-in	1.6±0.5%
Yb@$Lu_{0.8}Er_{0.2}$@Lu	Inside-out	0.4±0.2%
$Yb_{0.5}Lu_{0.4}Er_{0.1}$@Lu	Local energy transfer	0.6±0.2%
$Lu_{0.8}Er_{0.2}$@$Yb_{0.75}Lu_{0.25}$@Lu	Outside-in, interior shell	1.5±0.3%
$Lu_{0.8}Er_{0.2}$@$Yb_{0.5}Lu_{0.5}$@Lu	Outside-in, interior shell	1.3±0.3%
$Lu_{0.7}Er_{0.3}$@Yb@Lu	Outside-in	1.2±0.4%
Yb@$Lu_{0.7}Er_{0.3}$@Lu	Inside-out	0.3±0.1%
$Yb_{0.5}Lu_{0.35}Er_{0.15}$@Lu	Local energy transfer	0.5±0.2%
$Yb_{0.2}Lu_{0.78}Er_{0.02}$@Lu	traditional	0.7±0.4%

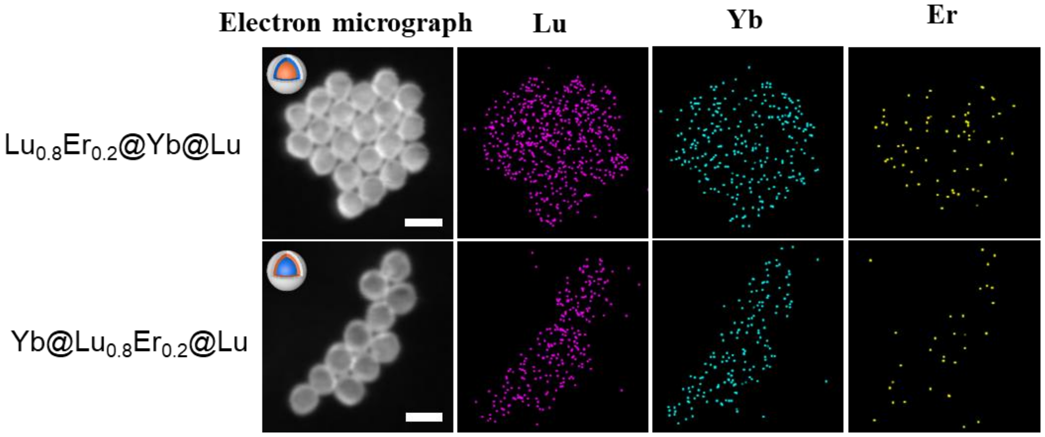

Supplementary Figure 3. SEM-EDS mapping images of outside-in and inside-out UCNPs with 20% Er^{3+} doping. These images were carried out in a FEI Tecnai G2 F20 X-TWIN TEM (at Fudan University). Three experiments were repeated independently with similar results. Scale bar, 20 nm.

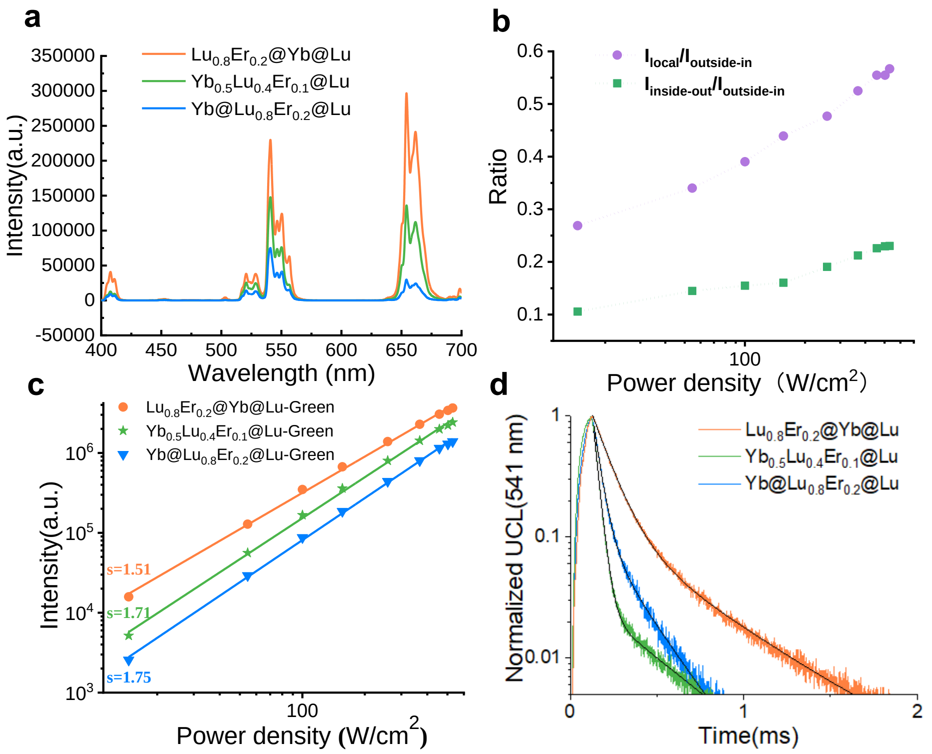

Supplementary Figure 4. Ensemble luminescence characterizations of outside-in $Lu_{0.8}Er_{0.2}$@Yb@Lu, inside-out Yb@$Lu_{0.8}Er_{0.2}$@Lu, and local $Yb_{0.5}Lu_{0.4}Er_{0.1}$@ Lu UCNPs. (a) UCL spectra of ensemble UCNPs in cyclohexane solution under 530 W/cm^2 980 nm laser excitation. **(b)** Power-dependence of intensity ratios. $I_{outside\text{-}in}$, $I_{inside\text{-}out}$, I_{local} represent the integrate photoluminescence intensities of $Lu_{0.8}Er_{0.2}$@Yb@Lu, Yb@$Lu_{0.8}Er_{0.2}$@Lu and $Yb_{0.5}Lu_{0.4}Er_{0.1}$@ Lu. **(c)** Power dependence of green emission at 541 nm. **(d)** Luminescence decay curves excited by 980 nm pulsed laser and recorded at 541 nm emission. Source data are available as Source Data file.

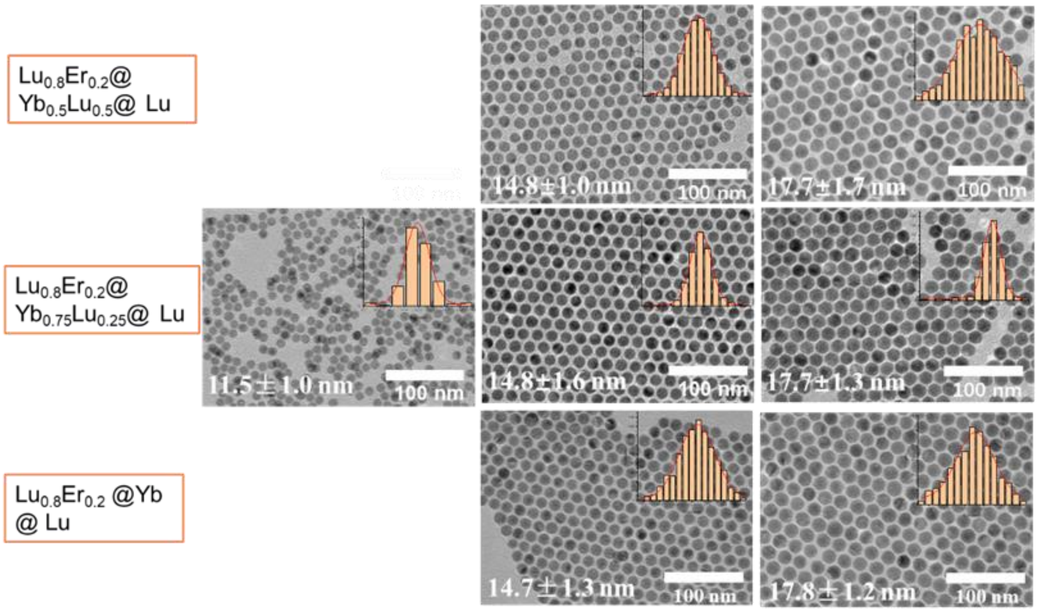

Supplementary Figure 5. TEM images of outside-in UCNPs with varied doping concentrations of Yb^{3+} ions in the interior layer. TEM images of core (left), core-interior shell (center) and final core-interior shell-inert shell UCNPs (right) with 50% (top), 75% (middle) and 100% (bottom) Yb^{3+} doping in the interior shells. Each panel includes a size distribution histogram with Gaussian fitting curve; the mean size (by Gaussian fitting) and standard deviation are shown in the lower left corner of each panel. More than five TEM images of each sample were included for statistical analysis, the results were presented as mean ± standard deviation.

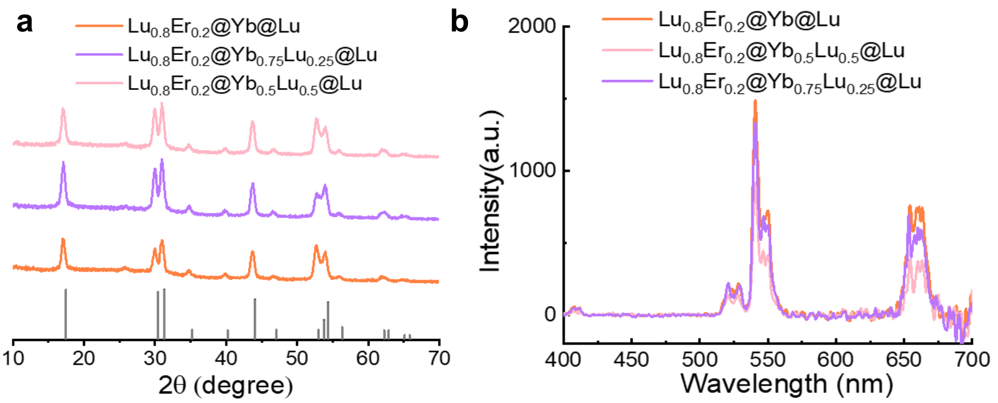

Supplementary Figure 6. Ensemble assessments by (a) XRD and (b) UCL spectra of outside-in UCNPs with varied doping concentration of Yb^{3+} ions in the interior layer. The XRD patterns were compared to standard spectrum of β-$NaLuF_4$ (#27-0726). The UCL spectra were obtained under 14.6 W/cm^2 980 nm laser excitation. Source data are available as Source Data file.

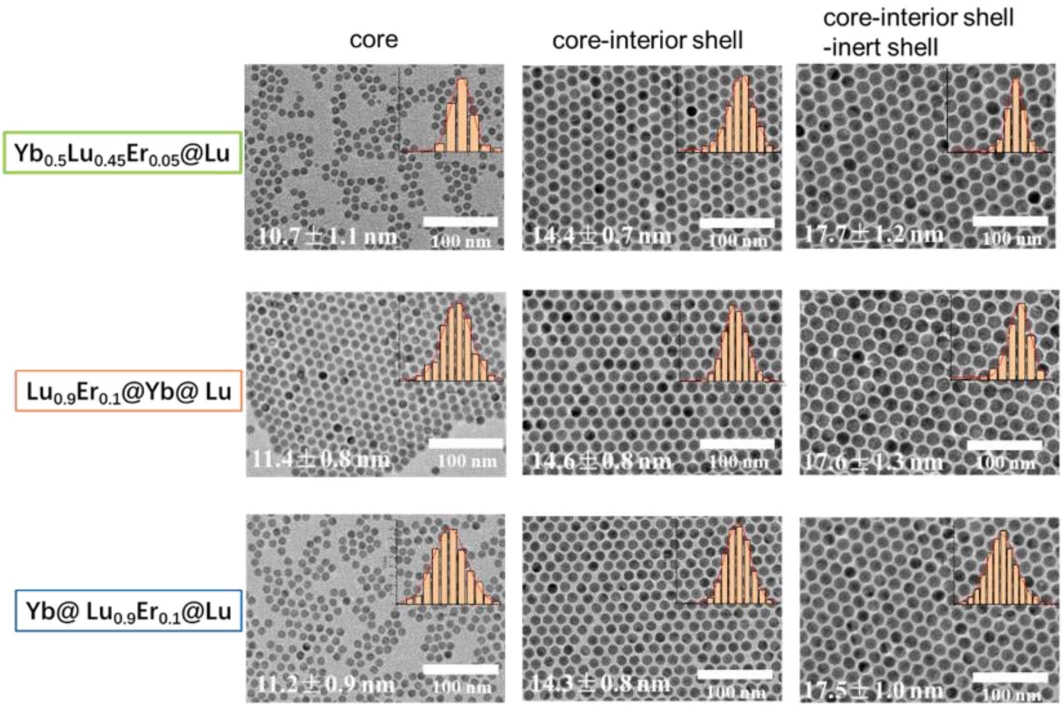

Supplementary Figure 7. TEM images of UCNPs with 10% Er^{3+} doping concentration. TEM images of core (left), core-interior shell (center) and final core-interior shell-inert shell UCNPs (right) in local (top), outside-in (middle) and inside-out(bottom) architectures. Each panel includes a size distribution histogram with Gaussian fitting curve; the mean size (by Gaussian fitting) and standard deviation are shown in the lower left corner of each panel. More than five TEM images of each sample were included for statistical analysis, the results were presented as mean ± standard deviation.

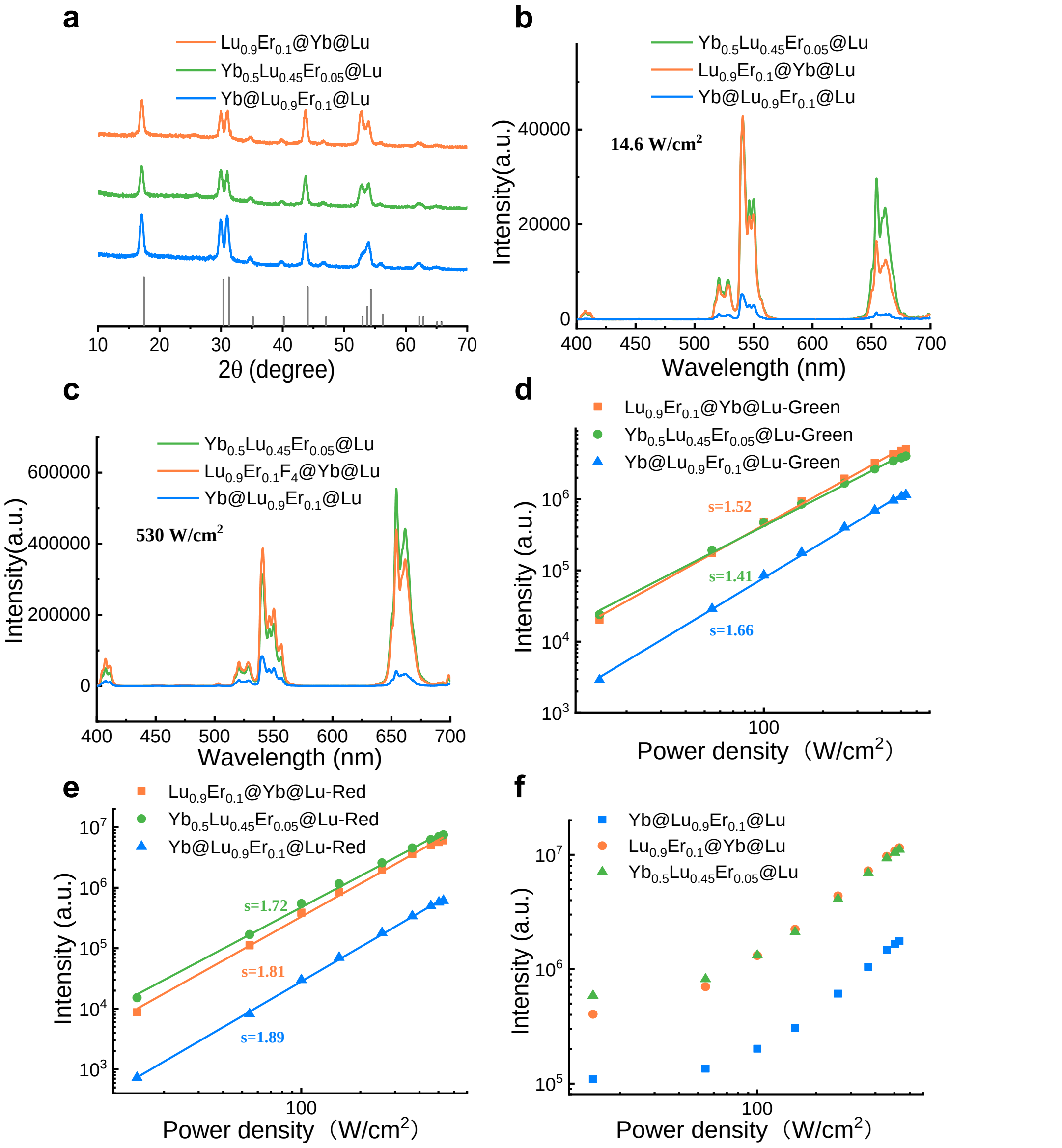

Supplementary Figure 8. Characterization of UCNPs with 10% Er^{3+} doping concentration. (a) XRD pattern of 10% Er^{3+} doped samples, compared with the standard spectrum of β-$NaLuF_4$ (#27-0726). **(b)** UCL spectra of ensemble UCNPs in cyclohexane solution under 14.6 W/cm^2 980 nm laser excitation. **(c)** UCL spectra of ensemble UCNPs in cyclohexane solution under 530 W/cm^2 980 nm laser excitation. **(d-e)** Power-dependence of green emission at 541 nm and red emission at 654 nm. **(f)** Power-dependence of integrated UCL emission from 400 to 700 nm. Source data are available as Source Data file.

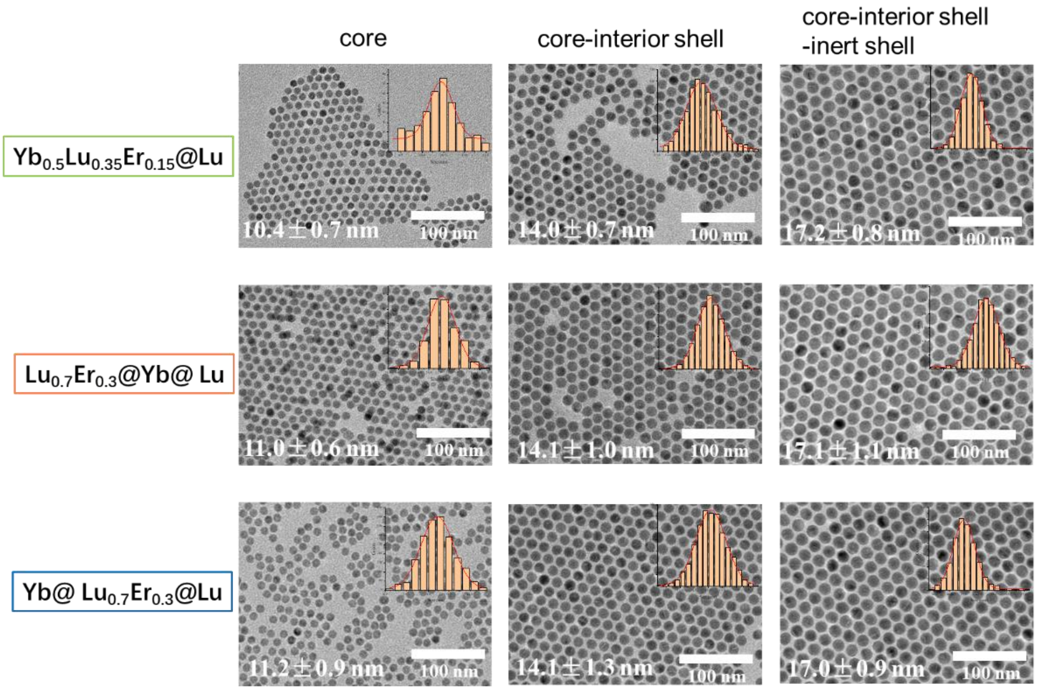

Supplementary Figure 9. TEM images of UCNPs with 30% Er^{3+} doping concentration. TEM images of core (left), core-interior shell (middle) and final core-interior shell-inert shell UCNPs (right) in local (top), outside-in (middle) and inside-out(bottom) architectures. The $NaYbF_4$ core for Yb@$Lu_{0.9}Er_{0.1}$@Lu was also used for preparing Yb@$Lu_{0.7}Er_{0.3}$@Lu. Each panel includes a size distribution histogram with Gaussian fitting curve; the mean size (by Gaussian fitting) and standard deviation are shown in the lower left corner of each panel. More than five TEM images of each sample were included for statistical analysis, the results were presented as mean ± standard deviation.

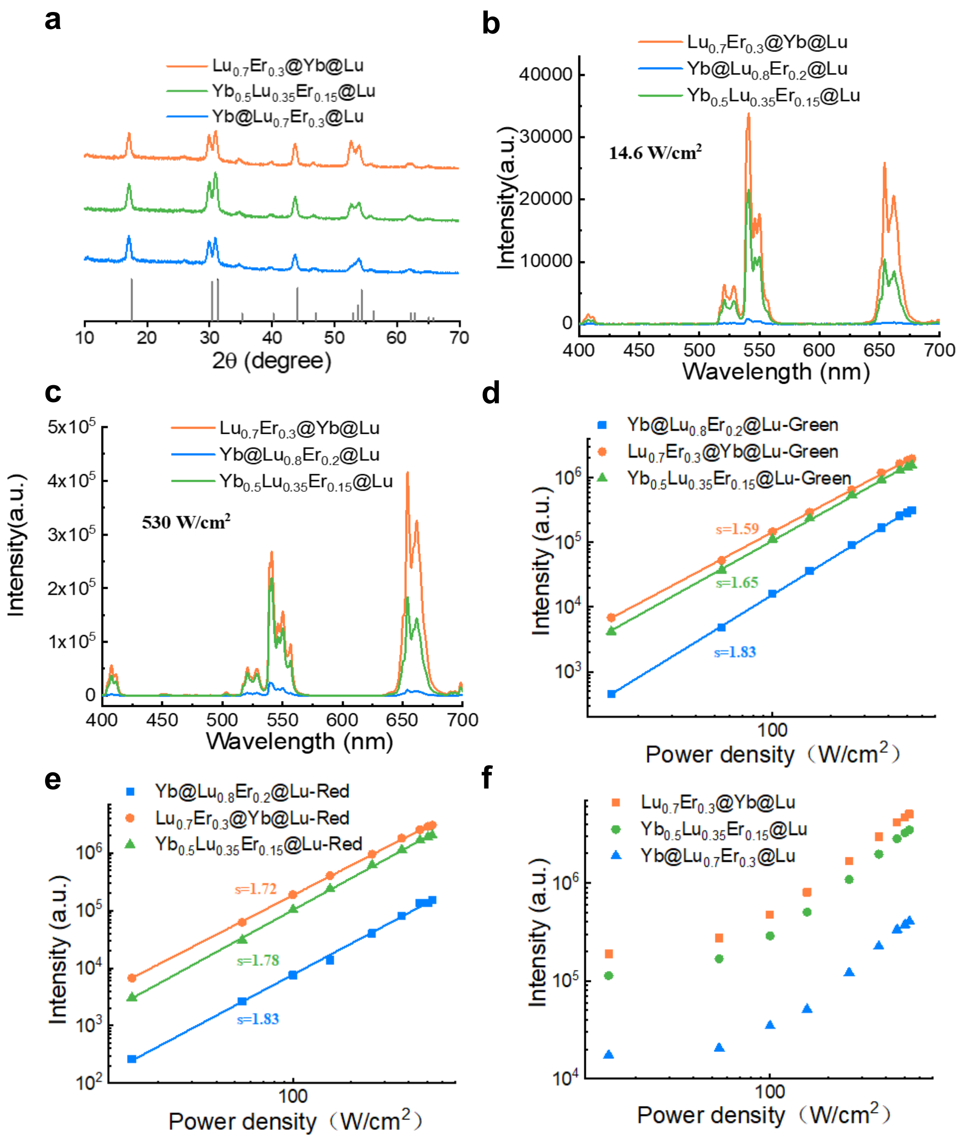

Supplementary Figure 10. Characterization of UCNPs with 30% Er^{3+} doping concentration. **(a)** XRD pattern of 30% Er^{3+} doped UCNPs, compared with the standard spectrum of β-$NaLuF_4$ (#27-0726). **(b)** UCL spectra of ensemble UCNPs in cyclohexane solution under 14.6 W/cm^2 980 nm laser excitation. **(c)** UCL spectra of ensemble UCNPs in cyclohexane solution under 530 W/cm^2 980 nm laser excitation. **(d-e)** Power-dependence of green emission at 541 nm and red emission at 654 nm. **(f)** Power-dependence of integrated UCL emission from 400 to 700 nm. Source data are available as Source Data file.

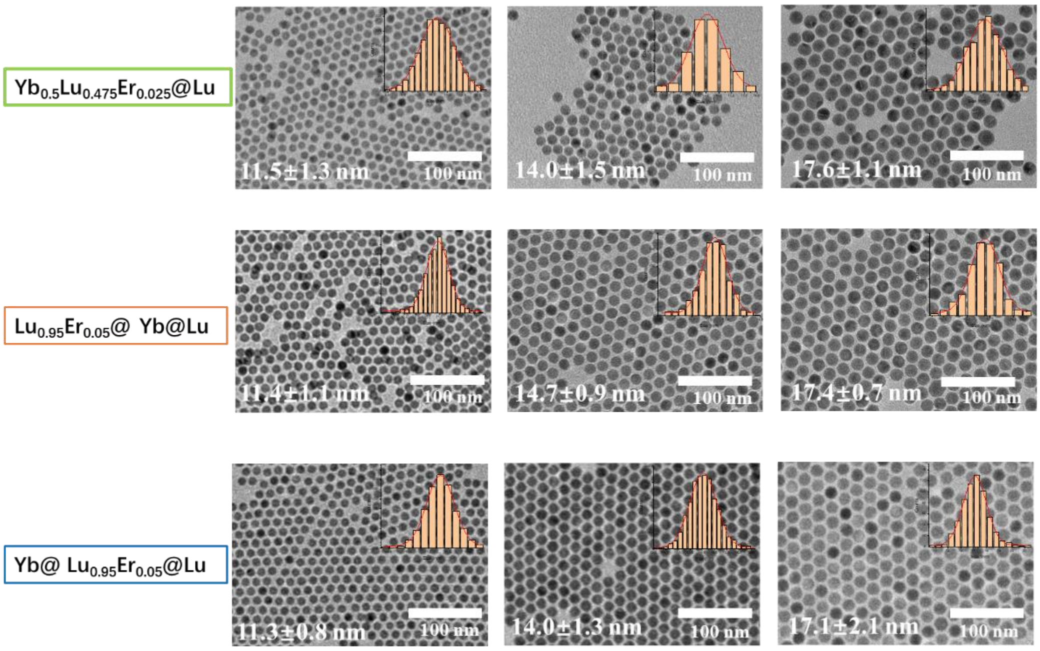

Supplementary Figure 11. TEM images of UCNPs with 5% Er^{3+} doping concentration. TEM images of core (left), core-interior shell (center) and final core-interior shell-inert shell UCNPs (right) in local (top), outside-in (middle) and inside-out(bottom) architectures. Each panel includes a size distribution histogram with Gaussian fitting curve; the mean size (by Gaussian fitting) and standard deviation are shown in the lower left corner of each panel. More than five TEM images of each sample were included for statistical analysis, the results were presented as mean ± standard deviation.

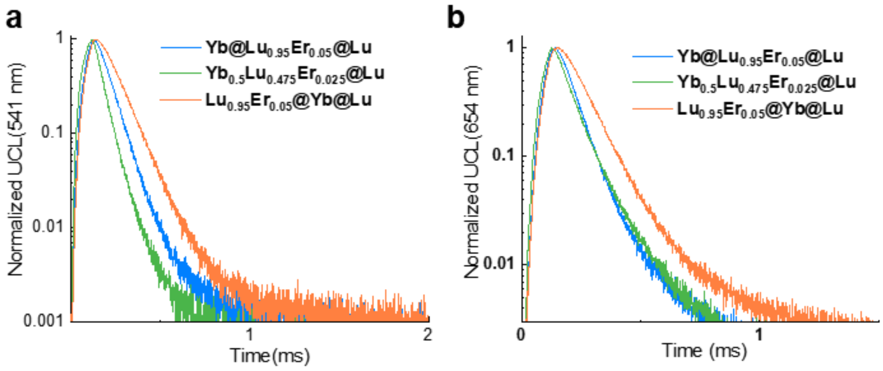

Supplementary Figure 12. Luminescence decay curves of UCNPs with 5% Er^{3+} doping concentration, excited by 980 nm pulsed laser and recorded at (a) 541 nm; (b) 654 nm emission. Source data are available as Source Data file.

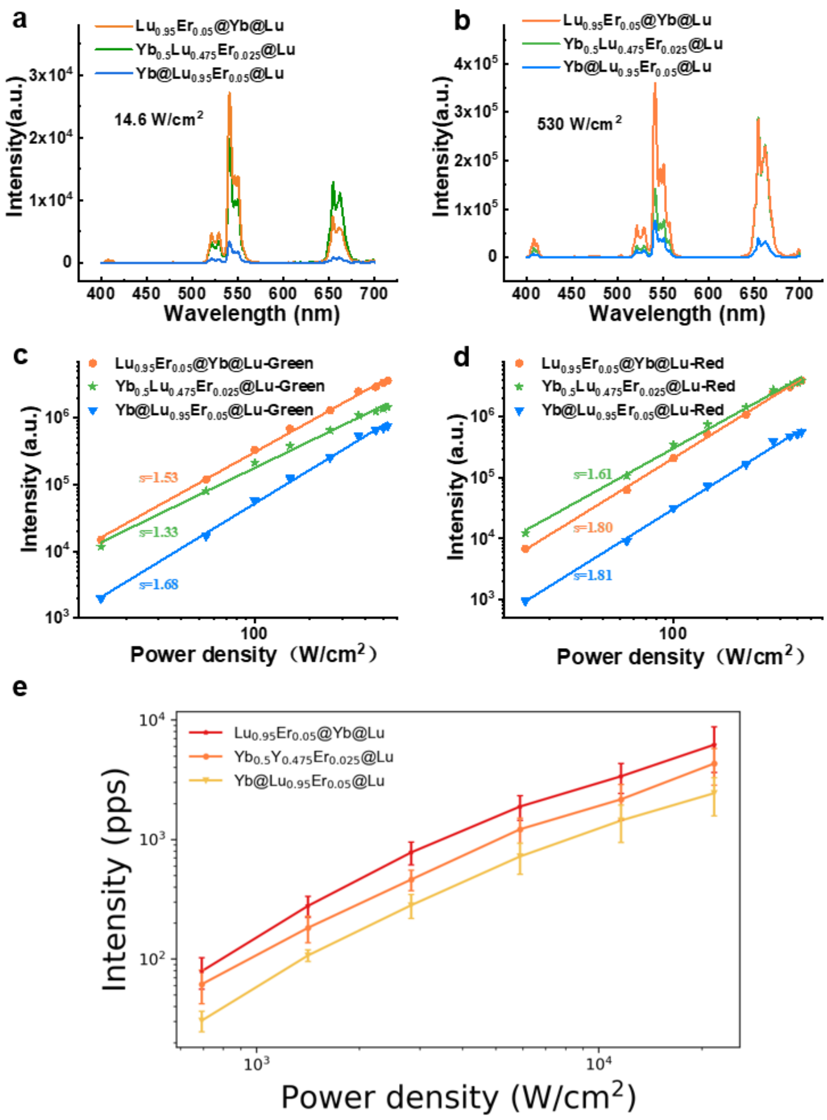

Supplementary Figure 13. Characterization of UCNPs with 5% Er^{3+} doping concentration. **(a)** UCL spectra of ensemble UCNPs in cyclohexane solution under 14.6 W/cm^2 980 nm laser excitation. **(b)** UCL spectra of ensemble UCNPs in cyclohexane solution under 530 W/cm^2 980 nm laser excitation. **(c-d)** Power-dependence of green emission at 541 nm and red emission at 654 nm. **(e)** Saturation curves of single particle brightness at power densities from 126 W/cm^2 to 21.7 kW/cm^2 obtained with wide-field microscopy. The results were presented as means ± standard deviation (2 independent experiments, more than 5 field of views wide-field images were acquired for each experiment; "n" represents the number of single nanoparticles, $Lu_{0.95}Er_{0.05}$@Yb@Lu: n=252, $Yb_{0.5}Lu_{0.475}Er_{0.025}$@ Lu: n=195, Yb@$Lu_{0.95}Er_{0.05}$@Lu: n=137). Source data are available as Source Data file.

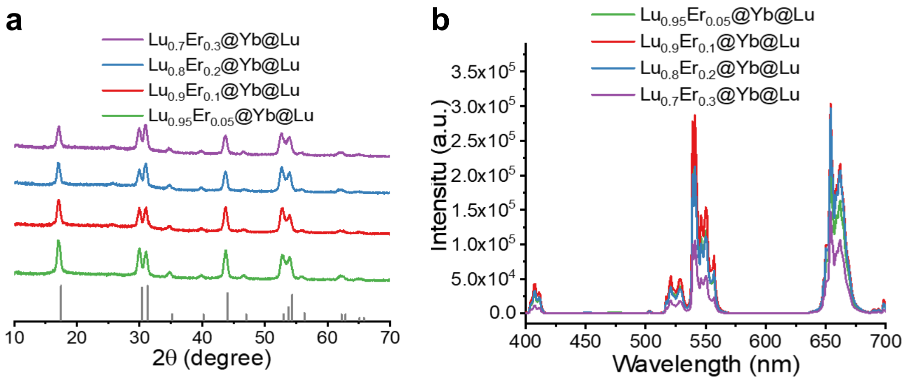

Supplementary Figure 14. Characterization of outside-in UCNPs with varied Er^{3+} doping concentration in the core. (a) XRD pattern of UCNPs with varied Er^{3+}, compared with the standard spectrum of β-$NaLuF_4$ (#27-0726). **(b)** Ensemble UCL spectra of UCNPs in cyclohexane solution under 530 W/cm^2 980 nm laser excitation. Source data are available as Source Data file.

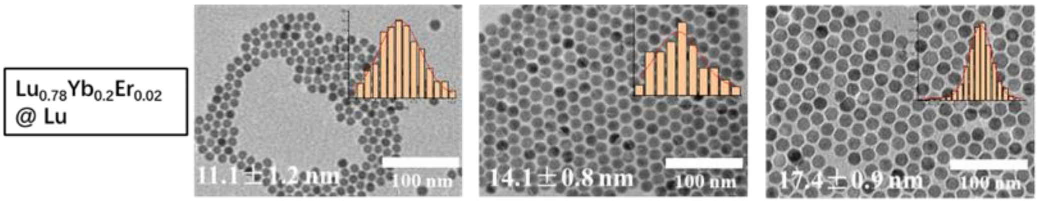

Supplementary Figure 15. TEM images of UCNPs of $Lu_{0.78}Yb_{0.2}Er_{0.02}$@ Lu. TEM images of core (left), core-interior shell (center) and final core- interior shell-inert shell UCNPs (right). Each panel includes a size distribution histogram with Gaussian fitting curve; the mean size (by Gaussian fitting) and standard deviation are shown in the lower left corner of each panel. More than five TEM images of each sample were included for statistical analysis, the results were presented as mean ± standard deviation.

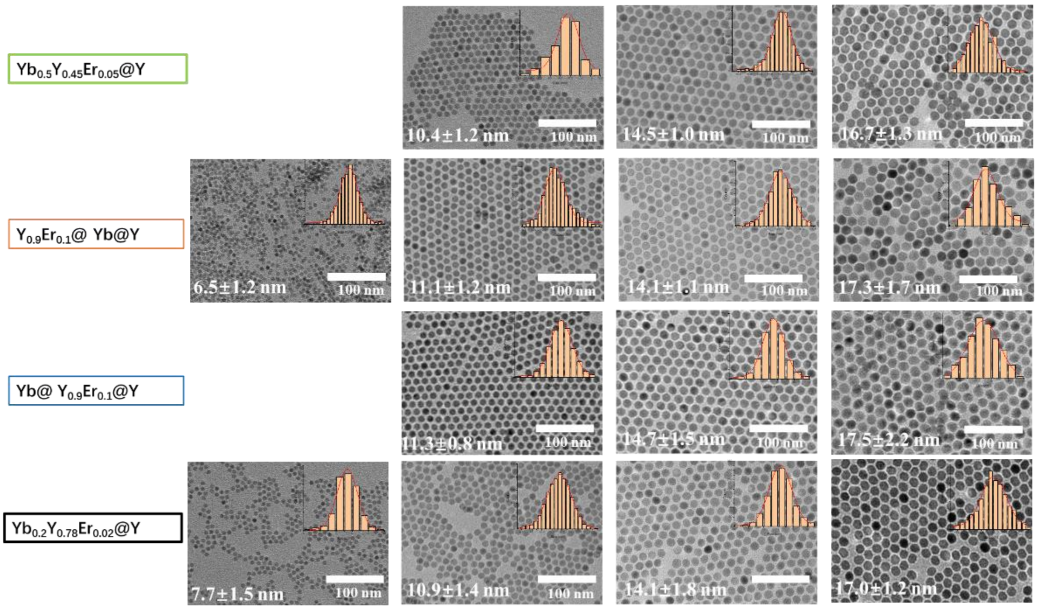

Supplementary Figure 16. TEM images of Y-based UCNPs with 10% Er^{3+} doping concentration. TEM images of ~7 nm core (left), ~11 nm core or core-interior shell (second column), ~14.5 nm core-interior shell (third column) or core-interior shell-outside shell (right), and final core-interior shell-inert shell or core-interior shell-outside shell-inert shell UCNPs (right) in local (top), outside-in (second row), inside-out (third row) and conventional (bottom) architectures. The $NaYbF_4$ core for Yb@$Lu_{0.95}Er_{0.05}$@Lu was also used for preparing Yb@$Y_{0.9}Er_{0.1}$@Y. Each panel includes a size distribution histogram with Gaussian fitting curve; the mean size and standard deviation are shown in the lower left corner of each panel. Highly doped Y^{3+}, such as in $NaY_{0.9}Er_{0.1}F_4$ and $NaYb_{0.2}Y_{0.78}Er_{0.02}F_4$, induced a reduction of the core size from 11 nm to 7 nm when using the same synthesizing protocol. In order to make a fair comparison, we grew another layer of $NaREF_4$ with the same composition of the in initial core to make the nanoparticles' size around 11 nm. More than five TEM images of each sample were included for statistical analysis, the results were presented as mean ± standard deviation.

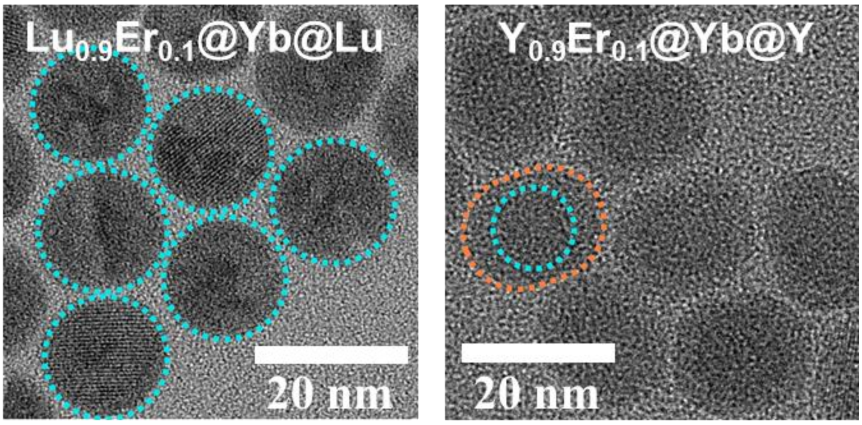

Supplementary Figure 17. The high-resolution TEM images of $Lu_{0.9}Er_{0.1}$@Yb@Lu (left) and $Y_{0.9}Er_{0.1}$@Yb@Y (right). The blue outlines represent the isotropy content with a near circle shape and the orange outlines represent the anisotropy $NaYF_4$ shell due to the lattice mismatch. More than five TEM images of each sample were recorded with similar characteristics.

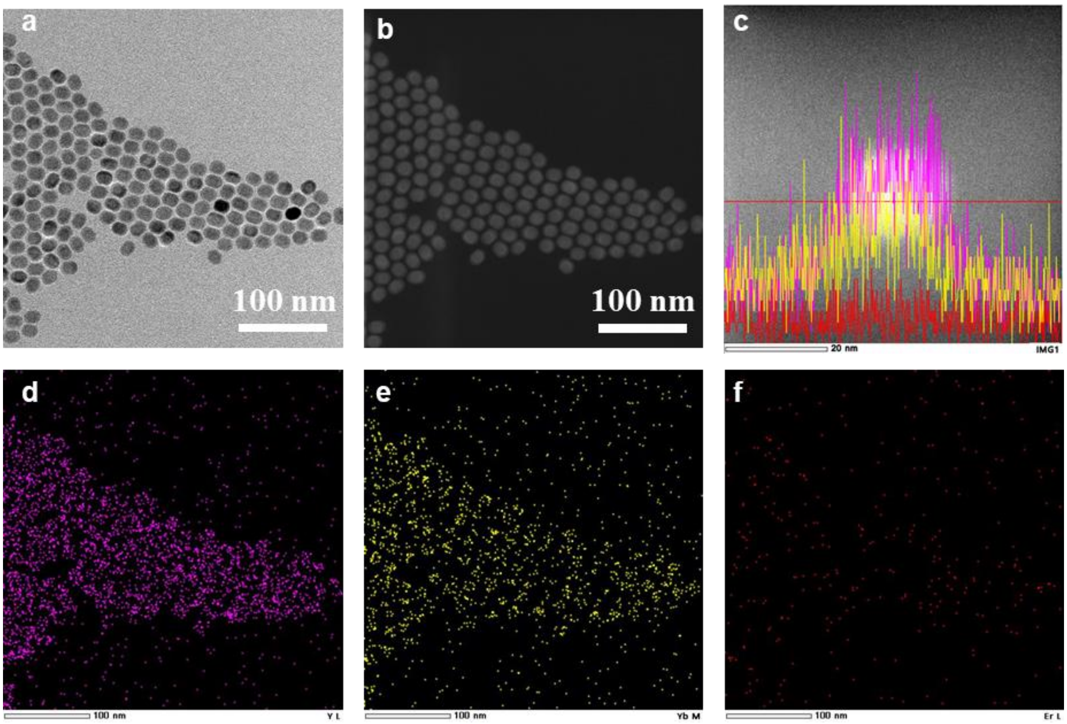

Supplementary Figure 18. **(a)** High-resolution TEM images, **(b)** High angle annular dark field (HAADF) scanning TEM (STEM) imaging of Y0.9Er0.1@Yb@Y. **(c)** Line scan of a single particle of $Y_{0.9}Er_{0.1}$@Yb@Y. **(d-f)** EDX mapping imaging corresponding to the same region with respect to (a-b). These images were carried out in a JEM-F200(URP) TEM (Shiyanjia Lab (www.shiyanjia.com)). Three experiments were repeated independently with similar results.

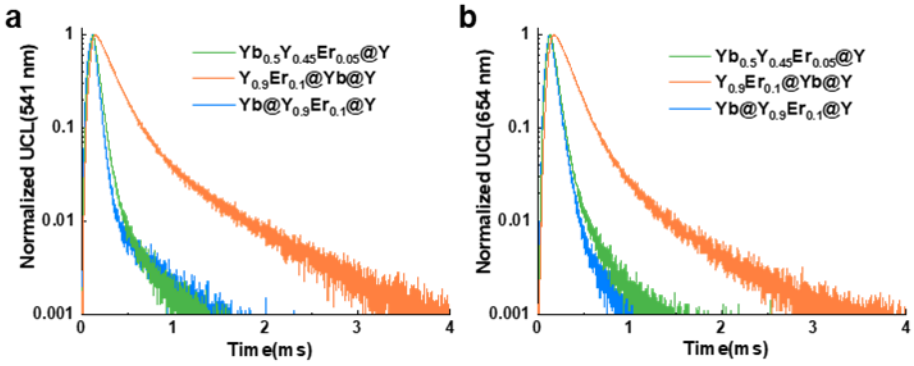

Supplementary Figure 19. Luminescence decay curves of Y-based UCNPs, excited by 980 nm pulsed laser and recorded at (a)541 nm; (b) 654 nm emission. Source data are available as Source Data file.

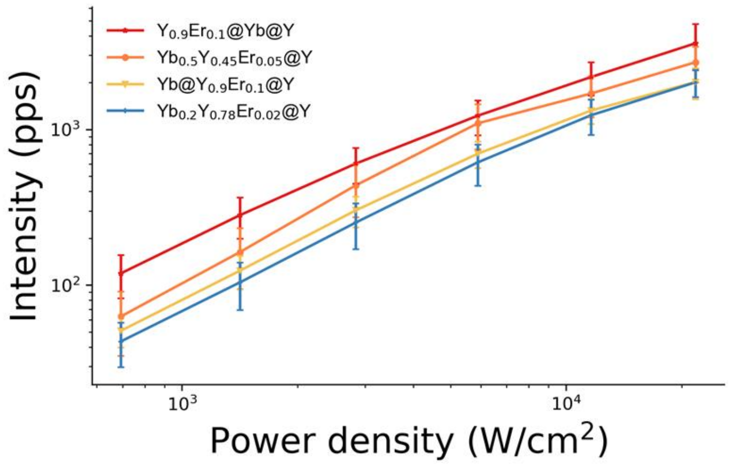

Supplementary Figure 20. Single particle imaging of Y-based UCNPs. Saturation curves of single particle brightness at power densities from 126 W/cm^2 to 21.7 kW/cm^2 obtained with wide-field microscopy. The results were presented as means ± standard deviation (2 independent experiments, more than 5 field of views wide-field images were acquired for each experiment; $Y_{0.9}Er_{0.1}$@Yb@Y: n=180, $Yb_{0.5}Y_{0.475}Er_{0.025}$@Y: n=224, Yb@$Y_{0.95}Er_{0.05}$@Y: n=274, $Yb_{0.2}Y_{0.78}Er_{0.02}$@ Y: n=186). Source data are available as Source Data file.

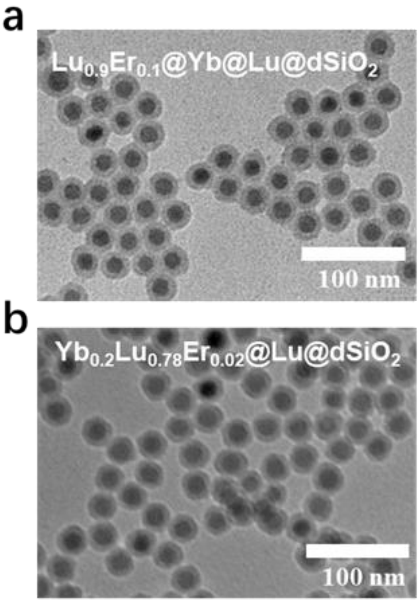

Supplementary Figure 21. TEM images of (a) $Lu_{0.9}Er_{0.1}$@Yb@Lu@dSiO_2 and (b) $Yb_{0.2}Lu_{0.78}Er_{0.02}$@Lu@d$SiO_2$. More than five TEM images of each sample were recorded with similar characteristics.

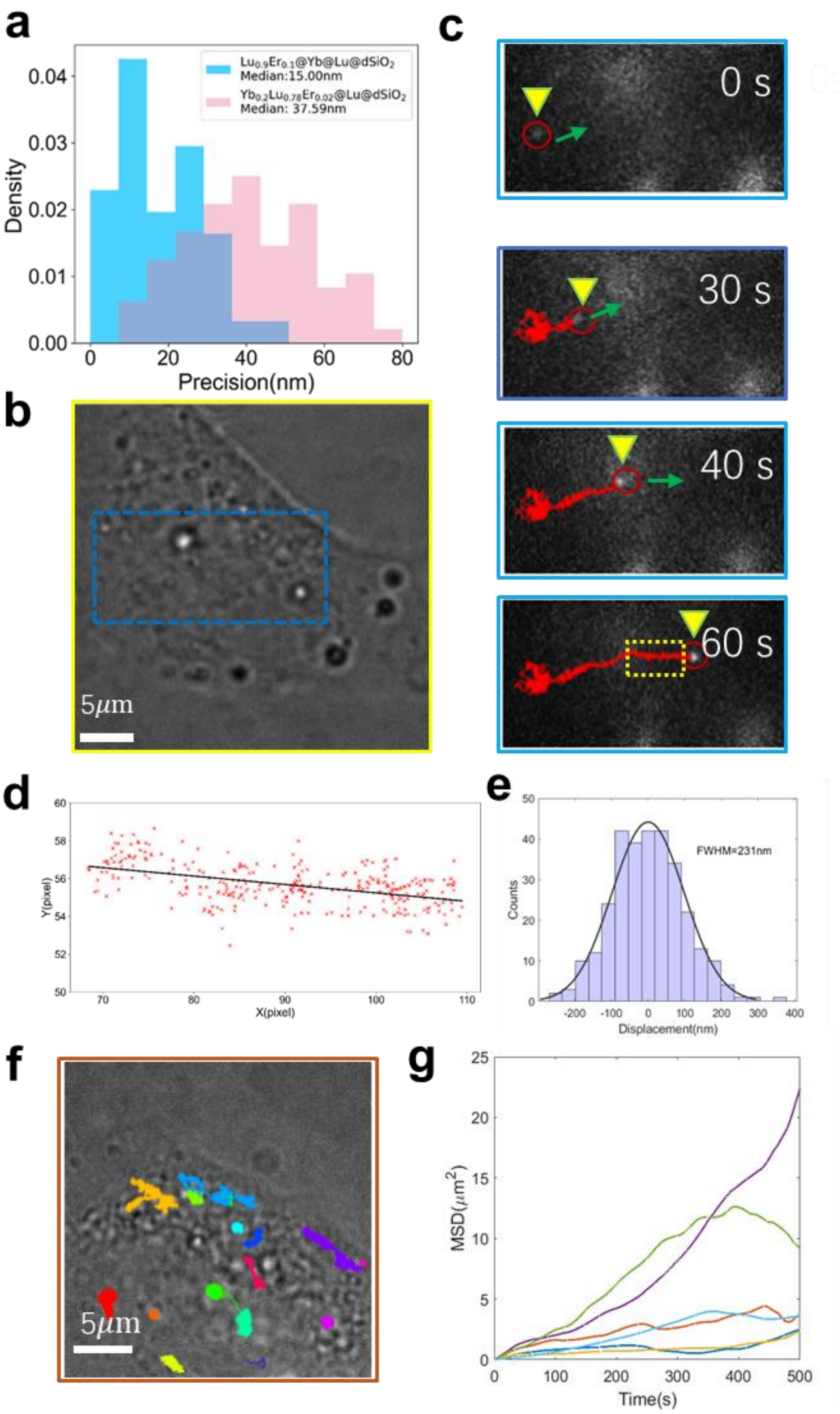

Supplementary Figure 22. Single particle tracking in living cells. (a) Localization precision frequency density distribution of $Lu_{0.9}Er_{0.1}$@Yb@Lu@dSiO_2 and $Yb_{0.2}Lu_{0.78}Er_{0.02}$@Lu@d$SiO_2$. **(b)** Bright field image of a U2OS cells loaded with $Lu_{0.9}Er_{0.1}$@Yb@Lu@dSiO_2. **(c)** Time-lapsed single particle images inside blue rectangle in (b) for a period of 60 secs with two particle trajectories overlaid, the direction is marked by the green arrows. **(d)** The part of trajectory distribution (yellow

box in **(c)**) and the fitted hypothetical line segment. **(e)** Deviation of the trajectory from the line segment. **(f)** $Lu_{0.9}Er_{0.1}$@Yb@Lu@dSiO_2 loaded U2OS cell with trajectories of particles ($n_{trajectories}$=16) moving randomly in different directions. **(g)** Representative MSD curves plot for these trajectories of (f). Three experiments were repeated independently with similar results. Source data are available as Source Data file.

Supplementary References:

1. Liu Q, Zhang Y, Peng CS, Yang T, Joubert LM, Chu S. Single upconversion nanoparticle imaging at sub-10 W cm^{-2} irradiance. *Nat. Photonics* **12**, 548-553 (2018).
2. Xiao Q*, et al.* A core/satellite multifunctional nanotheranostic for in vivo imaging and tumor eradication by radiation/photothermal synergistic therapy. *J Am Chem Soc* **135**, 13041-13048 (2013).
3. Chen X*,* et al. Confining energy migration in upconversion nanoparticles towards deep ultraviolet lasing. *Nat. Commun.* **7**, 10304 (2016).
4. Pertsinidis A, Zhang Y, Chu S. Subnanometre single-molecule localization, registration and distance measurements. *Nature* **466**, 647-651 (2010).
5. Fischer S, Johnson NJJ, Pichaandi J, Goldschmidt JC, van Veggel FCJM. Upconverting core-shell nanocrystals with high quantum yield under low irradiance: On the role of isotropic and thick shells. *Journal of Applied Physics* **118**, 193105 (2015).
6. Jones CMS, Gakamsky A, Marques-Hueso J. The upconversion quantum yield (UCQY): a review to standardize the measurement methodology, improve comparability, and define efficiency standards. *Sci Technol Adv Mater* **22**, 810-848 (2021).
