# Supplementary material for: Enhancement of single upconversion nanoparticle imaging by topologically segregated core-shell structure with inward energy migration
Source: Nat Commun. 2022 Oct 7;13:5927. doi: 10.1038/s41467-022-33660-8 (PMC9546905; doi:10.1038/s41467-022-33660-8)
Supplement: Supplementary file 3 — Description of Additional Supplementary [file 41467_2022_33660_MOESM3_ESM.docx]

Description of Additional Supplementary Files

File Name: Supplementary Movie 1

Description: Representative directional single-particle tracking based on Lu_0.9_Er_0.1_@Yb@Lu@dSiO_2_ in live U2OS cells.

File Name: Supplementary Movie 2

Description: Representative less directional single-particle tracking based on Lu_0.9_Er_0.1_@Yb@Lu@dSiO_2_ in live U2OS cells.
